# Decreased frontal white-matter diffusion and improved cognitive flexibility after burr-hole surgery in moyamoya angiopathy

**DOI:** 10.1186/s12883-020-1614-x

**Published:** 2020-01-20

**Authors:** Lionel Calviere, Paul Loubiere, Melanie Planton, Vanessa Cazzola, Isabelle Catalaa, Helene Mirabel, Jean Christophe Sol, Fabrice Bonneville

**Affiliations:** 1 Departments of Neurology, University Hospital of Toulouse, Toulouse, France; 2 Toulouse Neuro-imaging Centre, INSERM, University Paul Sabathier, Toulouse, France; 3 Department of Neurology, Hopital Pierre Paul Riquet, Place Dr. Baylac, 30159 Toulouse, France; 4 Department of Neuropsychology, University Hospital of Toulouse, Toulouse, France; 5 Department of Neuroradiology, University Hospital of Toulouse, Toulouse, France; 6 Department of Neurosurgery, University Hospital of Toulouse, Toulouse, France

**Keywords:** Apparent diffusion coefficient, Burr-hole surgery, Cognition, Cognitive flexibility, Moyamoya Angiopathy

## Abstract

**Background:**

In Moyamoya Angioplasty (MMA), increased apparent diffusion coefficient (ADC) in frontal white matter (WM) with a normal appearance has been associated with frontal hypoperfusion and executive dysfunction. Multiple burr-hole surgery enables the revascularization of large frontal areas.

Goal: To assess the effect of multiple burr-hole surgery on the ADC and cognitive functions in adults with MMA.

**Methods:**

ADC was measured in 26 brain hemispheres of 14 consecutive adults with MMA (9 women, mean age ± SD: 38.1 ± 10.7 years) prior to and 6 months after burr-hole surgery. ADC was obtained from regions of interest located in frontal and posterior (temporo-occipital) normal-appearing WM. Ten patients had neuropsychological assessment that focused on executive and attentional functions before and after surgery.

**Results:**

Anterior and posterior ADC values did not differ before surgery (815.8 ± 60.1 vs. 812.1 ± 35.3 mm^2^/s, *p* = 0.88). After surgery, frontal ADC was lower than prior to surgery (789.9 ± 64.5 vs. 815.8 ± 60.1 mm^2^/s; p <0.001) whereas no change occurred in posterior ADC (*p* = 0.31). Trail-making test part B median z-score increased from − 1.47 to − 0.21 (*p* = 0.018), suggesting improved cognitive flexibility.

**Conclusion:**

In adults with MMA, indirect revascularization with burr-hole is followed by a decrease of ADC in normal-appearing frontal WM and may have improved some executive functions in the flexibility process. Change in ADC may reflect the improvement in cerebral perfusion after surgery. The measuring of ADC may be a promising tool in exploring potentially reversible microstructural WM damage related to hypoperfusion and cognitive change in MMA.

## Introduction

Moyamoya Angioplasty (MMA) is characterised by progressive stenosis and occlusion of the intracranial carotid artery and its main branches, with the development of a basal collateral network [1]. These occlusive arterial lesions lead to chronic impairment of cerebral perfusion, often in frontal areas, resulting in stroke and cognitive disorders [2, 3]. Revascularization surgery is performed to improve cerebral perfusion and decrease the risk of stroke and death [4], but little is known about its effect with respect to other manifestations of MMA. Among the different surgical techniques available, there are several indirect revascularization techniques that are possible. Multiple burr-hole surgery is relatively easy to perform and allows the revascularization of large frontal areas via the temporal and middle meningeal arteries [5–7].

In cerebrovascular disease, increased apparent diffusion coefficient (ADC) in normal-appearing white matter (WM), as obtained by diffusion-weighted imaging (DWI), may be related to subtle structural changes caused by chronic hypoperfusion [8, 9]. In line with this, some studies have shown a correlation between increased ADC in normal-appearing WM and impaired cerebrovascular reserve (CVR) in MMA [10, 11]. However, there is no data on the evolution of ADC values after revascularization surgery.

A few studies have reported a high prevalence of cognitive disorders in adults with MMA. Among these, dysexecutive syndrome has been frequently mentioned [3, 12–16]. We have previously demonstrated an association between executive dysfunction, reduced frontal CVR and increased ADC in normal-appearing frontal WM [11, 12]. This finding was confirmed by other authors who also assessed ADC [17]. The relationship between WM diffusivity and cognition was also suggested in a study using diffusion tensor imaging (DTI) [18]. So, some cognitive disorders in MMA may be related to chronic hypoperfusion. The possibility that revascularization, specifically of the frontal areas, could change cognitive functioning has been only demonstrated in case reports or in limited series [19–22].

The aim of our study was to assess the evolution of ADC and its relationship with cognitive functioning after multiple burr-hole surgery in adults with MMA.

## Materials and methods

This retrospective study included consecutive adults with MMA treated in our tertiary hospital by burr-hole surgery between March 2008 and November 2015.

During this period, patients with predominant frontal hypoperfusion were treated by burr-hole surgery in our establishment. French guidelines do not impose a specific technique for the revascularization in adults with MMA [23]. Studies in children and a few in adults have suggested considering burr-holes as an option for Moyamoya patients [7, 24]. This technique allows for a large-scale revascularization of frontal areas, unlike other techniques that are more efficient for improving the perfusion of peri-sylvian parenchyma on lateral hemispheres. These studies reported a good outcome being defined as improvement in perfusion and low rates of recurrent ischemic stroke. Based on this data, it was a hospital decision to choose multiple burr-hole surgery as a first line of treatment for patients with predominant frontal hypoperfusion. Moreover, at that time, our surgeons had poor experience of other techniques. Patients with haemorrhagic presentation or more diffuse hypoperfusion were treated in another centre with more experience of direct or indirect by-pass.

The procedure was performed as follows: After coronal incision, the epicranium was exposed. In front of the hypoperfused region, an epicranial flap was prepared and burr hole was performed. The dura mater and arachnoid were carefully opened under microscope and the epicranial flap was deposited against the cerebral cortex. A median of 7 (range 4–9) burr-holes were performed through the frontal bone, in symptomatic hemisphere(s) (uni or bilateral) to cover a maximum of frontal hypoperfused parenchyma. Antiplatelet drugs were conserved during the perioperative period. Low molecular weight heparin was used 24 h after surgery to prevent venous thrombosis until patients had recovered sufficiently to be able to walk.

MMA was diagnosed using established criteria [25]. Each patient had undergone Digital Subtracted Angiography (DSA), DWI/ perfusion-weighted images (PWI) MRI, and neuropsychological assessment before and at 6 months after surgery.

Baseline demographic, clinical, and radiological data were recorded in our database. The study was approved by our medical establishment review board. In France, patient consent is not needed for retrospective clinical studies using data of standard care.

MRI studies were performed on a 3.0-T scanner (Skyra, Siemens, Germany). DWI, T2-weighted imaging (T2), Fluid-Attenuated Inversion Recovery (FLAIR), T2 Gradient Echo (T2 GRE), Gadolinium-enhanced T1-weighted imaging (T1 G), and circle of Willis Time Of Flight Magnetic Resonance Angiography (TOF) were acquired. PWI were acquired before and after IV injection of acetazolamide (13 mg/kg).

### Measurement of ADC

DWI were acquired using a spin-echo single-shot echo-planar sequence with the following parameters: Repetition Time (TR): 7410, Echo Time (TE) 72 ms, Field of View (FOV) 100, matrix, “132 x 132”, flip angle “180”, 3-mm-thick contiguous sections, Excitations Number (NEX) “1”, b = 0--1000 mm^2^/s. Three gradient directions were used. DWI were transferred to a separate workstation for data analysis. ADC maps were automatically generated voxel by voxel. Values were extracted from theses maps within region of interest (ROI).

**Fig. 1:**
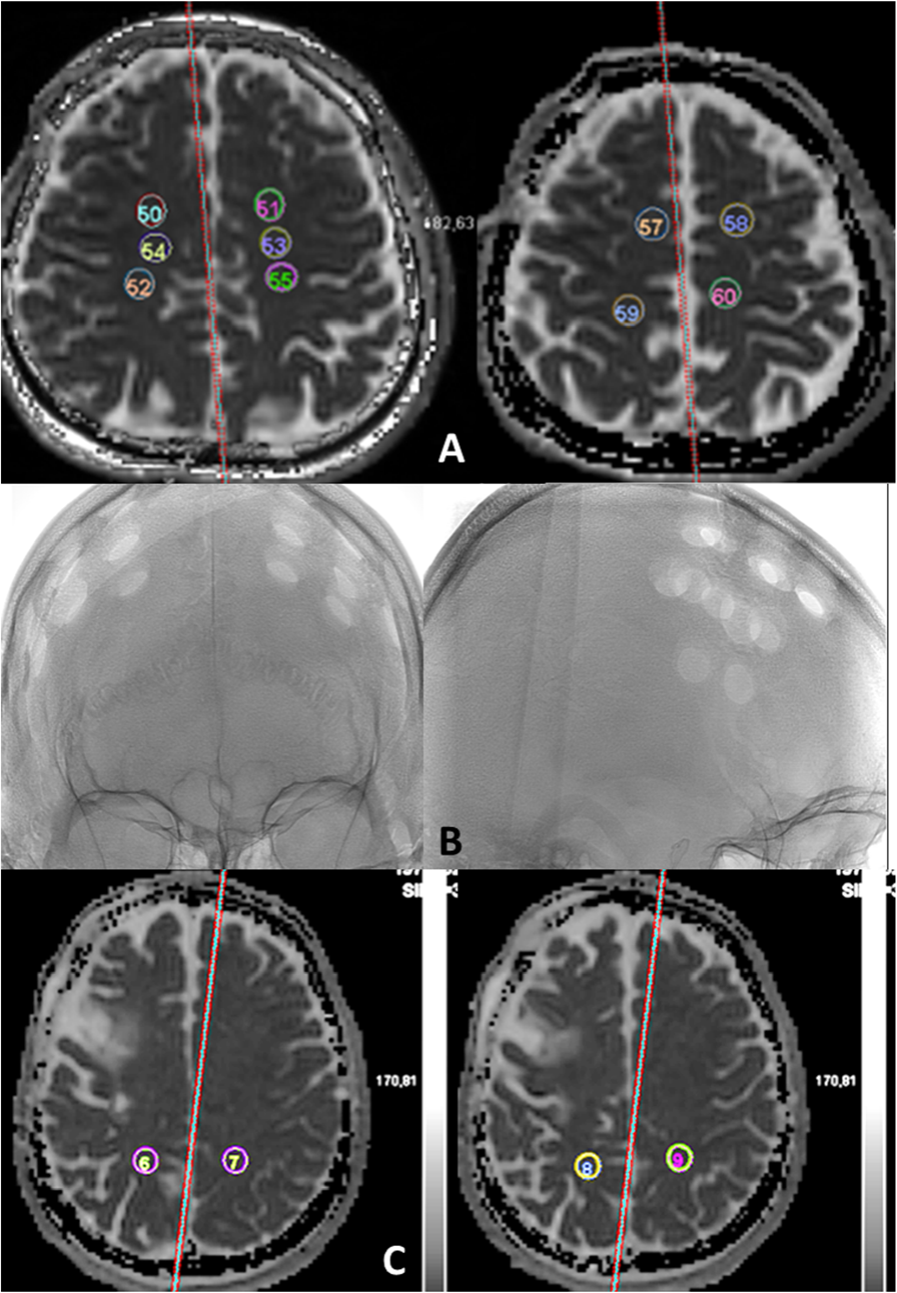
Template of the location of regions of interest (ROI) in normal-appearing white matter (Anterior ROIs (**a**); posterior ROIs (**c**) with an example of burr-holes location on angiography (**b**) to show that posterior ROIs were far from burr holes). ADC sequences passing through frontal white matter at the level of centrum semiovale. A minimum of 4 ROIs were placed in each hemisphere, but if possible, 1 or 2 more ROIs were added. The template was copied after surgery

In each surgical hemisphere, anterior ADC was calculated from an average of at least four ROI located in normal-appearing frontal WM. Each region of interest consisted of a 1-cm-diameter circle. We carefully avoided infarctions or leukoaraiosis as identified on T2WI and FLAIR. On the MRI carried out before surgery we drew a template of multiple ROIs placed on the 2 same slices passing through frontal WM at the level of centrum semiovale (Fig. 1). Templates were placed accurately by eye according to anatomical landmarks. This baseline template was the same for each patient and then adapted individually, if needed, by moving any ROI out of ischemic lesions and WM hyperintensities. A minimum of 4 ROIs were placed in each hemisphere, but if possible, 1 or 2 more ROIs were added. For each patient, the initial template obtained in this way was then copied on the MRI performed after surgery in order to have the same number of ROIs at exactly the same locations for each individual patient. To define control areas, regions of interest were placed in the normal-appearing posterior (temporo-occipital) WM, that was supposed to be less affected by MMA and burr-hole surgery. Exactly the same method was used with another template of ROIs placed posteriorly in the cerebral WM, on the same slices, to obtain a mean posterior ADC. All regions of interest were assessed by two neuroradiologist investigators (PL and VC) who were blind to the clinical data. The reproducibility between the two investigators was acceptable with a coefficient of concordance correlation of 0.77 (95% confidence interval (0.63–0.86); p <0.001). The same investigators (PL and VC) confirmed the absence of signal change on T2 / FLAIR.

PWI were acquired using a gradient echo sequence with the following parameters: TR: 1710, TE 20 ms, FOV 100, matrix, “108 x 108”, flip angle “90°”, 4-mm-thick contiguous sections, NEX “1”. We measured cerebral perfusion using dynamic susceptibility contrast-enhanced MRI. Gadolinium (DOTAREM; Guerbet, Villepinte, France) was injected twice at a rate of 5 ml/sec and a dose of 0.2 cc/kg. PWI, with measurement of relative regional CVR using the cerebellum as a reference region, was assessed as previously described [12]. In summary, we measured cerebral blood volume in each hemispheric region of interest and two reference regions of interest in the cerebellum. We calculated relative regional CVR using the regional cerebral-blood volume ratio before and after acetazolamide.

### Neuropsychological assessment

Neuropsychological tests focused on executive and processing speed functions included the Trail Making Test (Part A & B) for flexibility process, the Stroop test for inhibition, the Modified Card Sorting Test for conceptualization, and verbal fluency for initiation [26]. *The tests were performed by two experienced neuropsychologists (HM and MP) from our department of neuropsychology, blind to MRI data.* The patients’ individual cognitive test scores were converted to *z*-scores using the means and standard deviations (SDs) of clinical norms.

We defined significant improvement after surgery as at least two tests with Z-scores that had increased by 1 SD or become greater than − 1 SD (normal range) following revascularization. A decline was defined by at least two tests with Z-scores that had decreased by 1 SD.

### Collateral assessment

In our centre, 6-axis DSA is performed before and 6 to 9 months following surgery, to more effectively assess the arterial lesions and existing anastomoses initially, and further development of pial and transdural anastomoses after surgery. Collateral grades were assessed using the Matsuchima scale in middle and anterior cerebral arteries territories [27].

### Statistical analysis

We considered each hemisphere separately as an independent parameter, as is usually the case in MMA. Because of the small number of subjects and the abnormal distribution, we performed non parametric tests.

We compared WM ADC values (frontal and posterior) and CVR calculated from MRI PWI taken pre- and post- surgery, using Wilcoxon’s test.

The cognitive scores of MMA patients with major surgical complication or severe aphasia at the time of the neuropsychological assessment (*n* = 2) were not taken into account in the comparative analysis. We also excluded cognitive scores from MMA patient with major cognitive disorders before surgery (i.e., dementia) (n = 2). We used Wilcoxon’s test to compare neuropsychological *z*-scores taken before and after surgery.

Correlation between neuropsychological tests and ADC change before and after surgery was assessed with Pearson correlation test. For ADC, we used the average of the both hemispheres in each patient. We compared the ADC change between patients with neuropsychological improvement and those without, using Mann Whitney’s test.

Values are given as means ± SD except where specified. All tests were bilateral. A *p*-value < 0.05 was considered statistically significant. The Bonferonni correction was applied to anterior ADC comparisons (before versus after surgery and anterior versus posterior ADC). The level of significance became < 0.025. Statistical analysis was performed using SPSS 14.0 software (IBM analytics, IBM France Bois-Colombes).

## Results

### Study population

Fourteen patients (9 women, 5 men: mean age 38.1 ± 10.7 years) were included in this study. Ethnicities were Caucasian (*n* = 8), North African (*n* = 3), Asian (*n* = 2), and African--West-Indian (*n* = 1). Five patients had moyamoya syndrome: one case of sickle-cell disease, two had MMA associated with atheromatous lesions, one case of genetic malformative syndrome, and one of arterial dysplasia with dissections and MMA. The arterial lesions were bilateral in 12/14 patients. MMA was revealed by ischemic stroke in 11 patients, headache with a cognitive disorder in two and a transient ischemic attack in one patient. Twelve patients had at least one ischemic lesion when a MRI was performed.

Burr-hole surgery was decided on due to:

- symptomatic disease (ischemia (*n* = 12) or headache associated with cognitive disorders (*n* = 2))
- and predominant frontal hypoperfusion with impaired fontal vascular reserve on MRI.

Twenty-six hemispheres were operated. The median durations (IQR 25--75%) of disease from the earliest symptom until surgery and from diagnosis until surgery were, respectively, 541 (219--935) and 265 (104--688) days. Eighty percent of hemispheres developed collaterals following surgery: 7.6% grade A, 23% grade B, and 50% grade C, according to the Matsushima scale [27] . Two perioperative infarctions occurred in two patients. After this period, no stroke recurrence was observed after 6 months of follow-up.

**Fig. 2:**
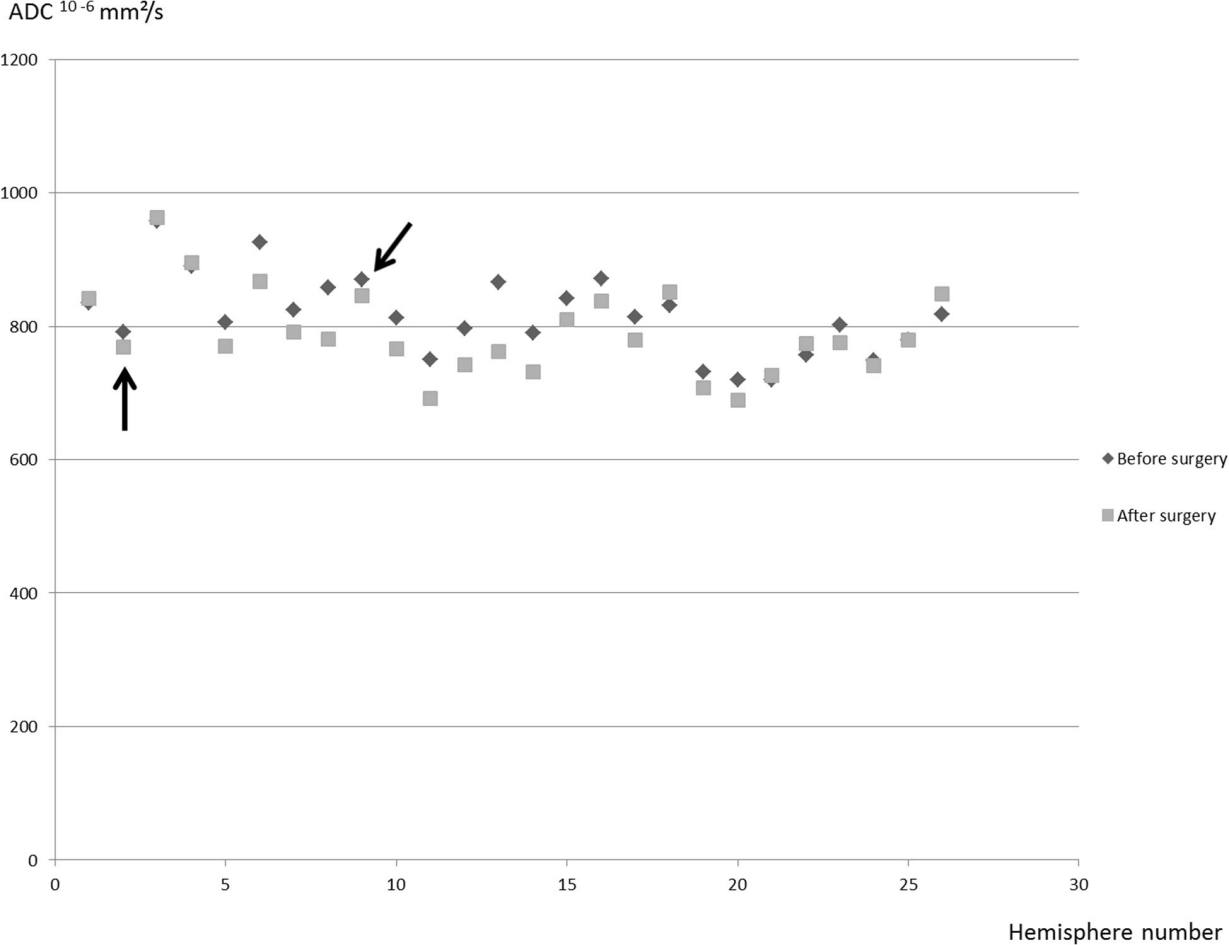
Scattering of the ADC values before (black) and after (grey) surgery for each hemisphere. Black arrows correspond to the 2 hemispheres with cortical infarctions related to surgery. The white matter ADC value also decreased after surgery. The values remained in the range of other hemsipheres

**Fig. 3:**
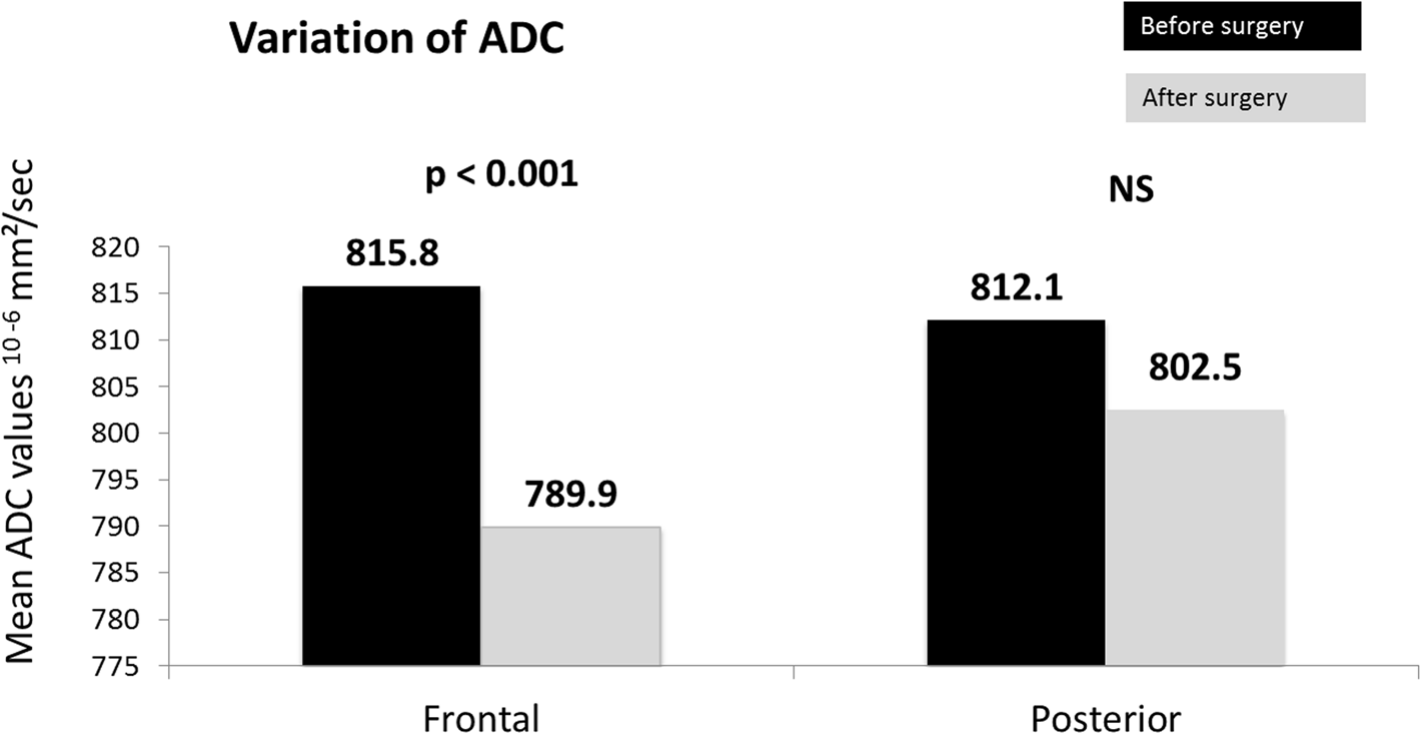
Comparison of frontal and posterior ADC values before and after burr-hole surgery. Significantly decreased (**p <0.001) ADC in frontal areas, and no significant changes to posterior areas

Since we considered each hemisphere separately as an independent parameter, 26 frontal ADC and 26 posterior ADC values were compared before and after surgery in our 14 patients (Fig. 2). Before surgery, there was no difference between frontal and posterior ADC values (815.8 ± 60.1 vs. 812.1 ± 35.3^10–6^ mm^2^/s, *p* = 0.88). After surgery, ADC value decreased in 19/26 (73.1%) hemispheres. Frontal ADC was lower than before surgery (789.8 ± 64.5 vs. 815.8 ± 60.1^10–6^ mm^2^/s; p <0.001) whereas no significant change was observed in posterior ADC (812.1 ± 35.3 vs. 802.04 ± 34.910^− 6^ mm^2^/s; *p* = 0.31 (0.31) (Fig. 3).

Including MMA with atherosclerosis as moyamoya syndrome is sometimes controversial. We performed an analysis without the 2 atherosclerotic patients. The results were similar. Frontal ADC was lower than before surgery (800.4 ± 63.8 vs. 818.4 ± 62.6 ^10–6^ mm^2^/s; *p* = 0.005) whereas no significant change was observed in posterior ADC.

PWI could be interpreted in only 24 hemispheres because of movements’ artefacts in one patient. Nevertheless, there was no artefact on the ADC maps in this patient. After surgery, no significant modifications were observed in the frontal or sub-cortical CVR (*p* = 0.54), for the entire population.

### Neuropsychology assessment

Clinically, the most impaired processes were flexibility (TMTB error and time, z-score = − 2.10 ± 2.2), conceptualization (number of categories in the modified Card Sorting Test, z-score = − 2.20 ± 3.3) and inhibition (Stroop test, interference score time, z-score = − 2.25 ± 4.5).

After surgery, six patients (60%) had cognitive improvement and two (20%) had a decline, according to our definition. Two patients had no significant change.

One patient with perioperative infarction had cognitive improvement. The other patient with perioperative infarction suffered from a large left stroke in the middle and anterior cerebral arteries. Due to severe aphasia and asthenia, he was not able to undergo neuropsychological assessment at six months and was one of the patients excluded from neuropsychological analysis.

**Fig. 4:**
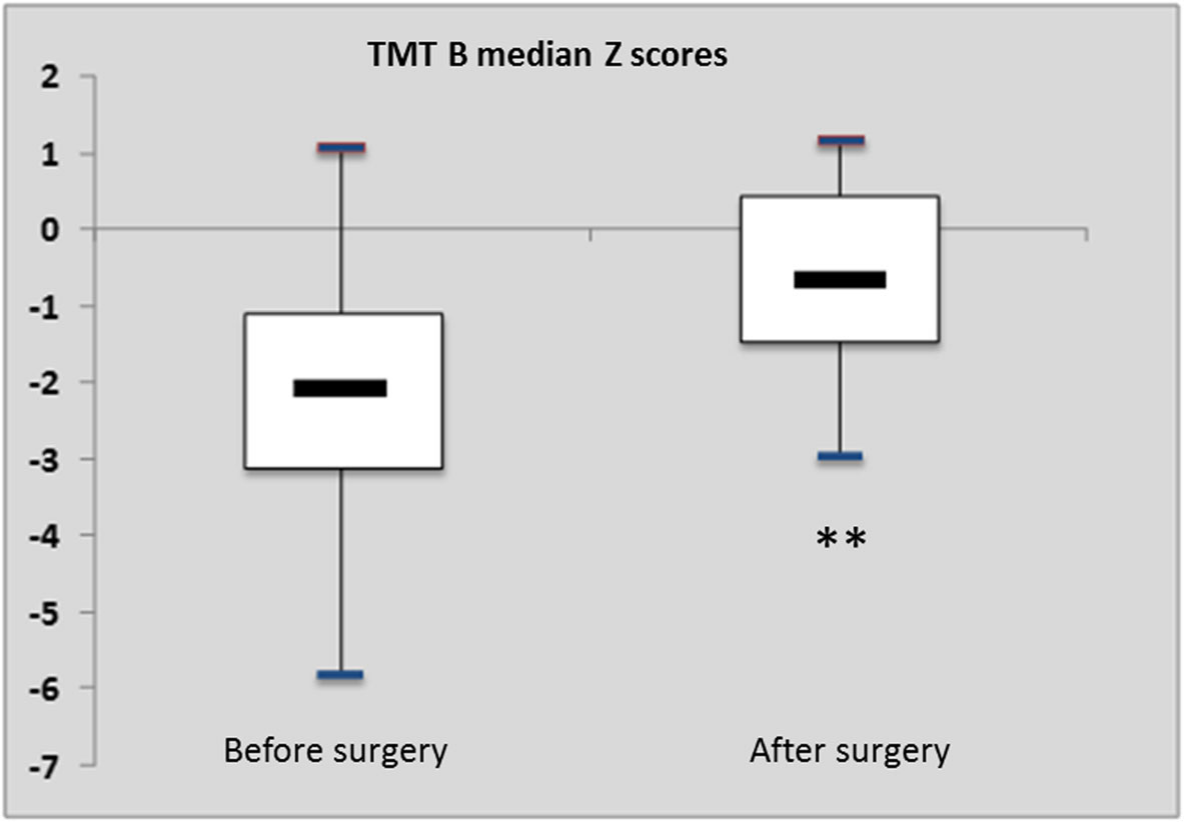
Box plots. TMT-B median *z*-score (IQR) increased from − 1.47 (− 3.1; − 1.1) before surgery to − 0.21 (− 1.5; 0.5) after surgery (*p* = 0.018 **)

**Fig. 5:**
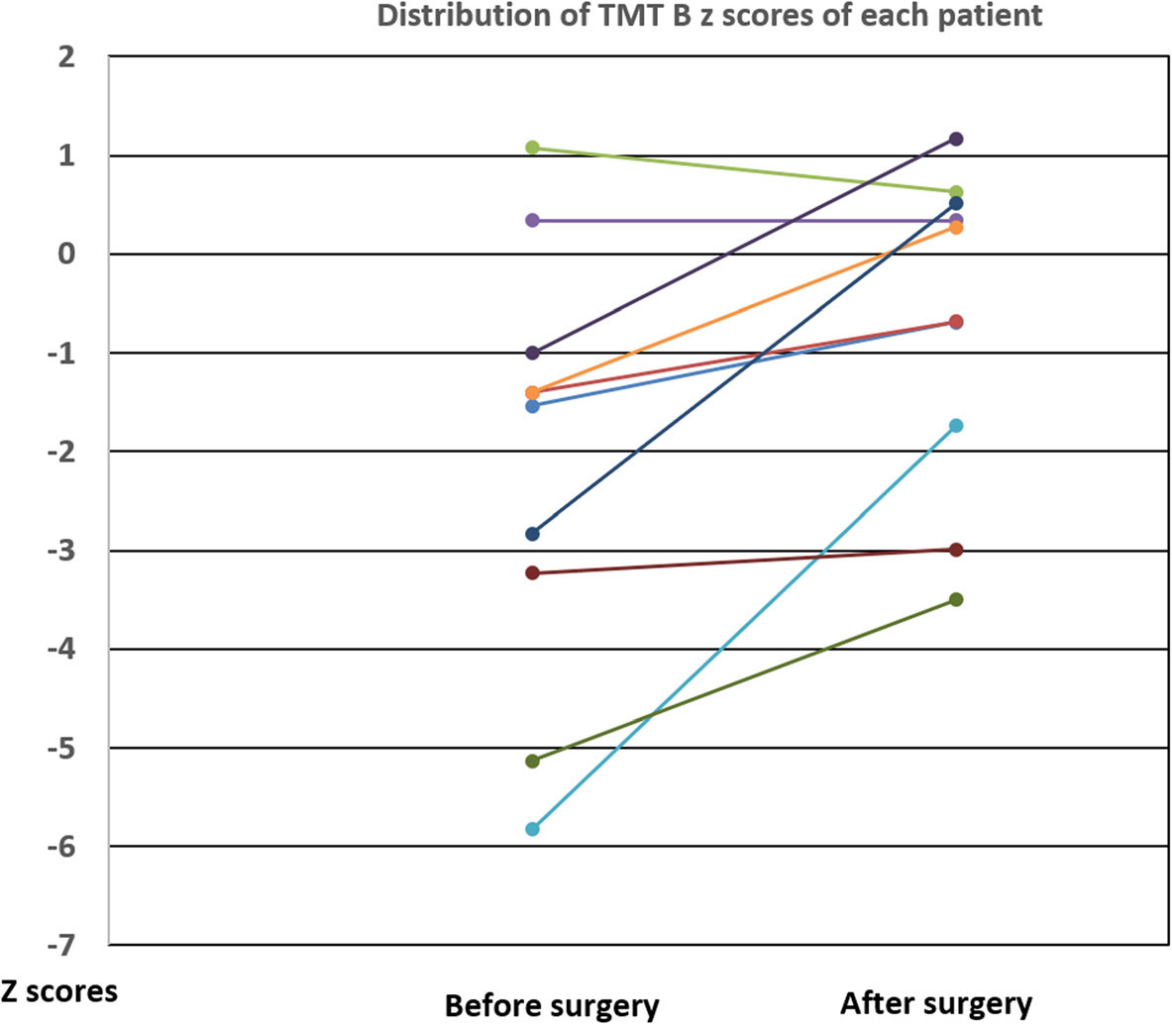
Representation of the z scores of each patient before and after surgery

TMT B median z-score (IQR) increased from − 1.47 (− 3.1; − 1.1) to − 0.21 (− 1.5; 0.5); *p* = 0.018 (Fig. 4). Figure 5 shows the distribution of the z-scores of each patient before and after surgery. No significant change was detected when comparing *z*-scores for other cognitive processes.

No significant correlation was observed between TMT B and ADC change (*p* = 0.5).

ADC values did not differ between patients with (*n* = 6) and without (*n* = 4) cognitive improvement (*p* = 0.55) or between patients with increased TMTB of at least 1 SD and the others (*p* = 0.9).

## Discussion

The primary finding of our study is that ADC values in frontal normal-appearing WM decreased after indirect revascularization with multiple burr-holes in adults with MMA as compared to the value prior to surgery. These values tended to normalise but remained increased as compared with healthy subjects of other series (around 700 ^10–6^ mm^2^/s). Previous studies have demonstrated a correlation between increased ADC in normal-appearing WM and hemodynamic disturbances in patients with MMA [10, 11] or cervical carotid stenosis [9]. Revascularization of frontal areas was the only operation performed on our patients. This led to the development of collaterals in 80% of hemispheres, with better visualization of frontal parenchymography on DSA. Therefore, the decrease in ADC was likely caused by frontal reperfusion. This relationship was reinforced by the absence of ADC modification in posterior areas that were unaffected by revascularization. Posterior ROIs were located far from burr-holes, in order to have control ROIs. This result was similar to previous work on carotid stenosis [28]. The authors reported higher WM ADC values in hemispheres ipsilateral to stenosis as compared to contralateral hemispheres. Following endarterectomy, ADC values decreased to the same level as those in the contralateral hemisphere. Moreover, some studies showed potential reversibility in WM lesions after direct or indirect surgery in patients with MMA. A case report described a decrease of ADC [29, 30]. Our work agrees with these findings, as some authors consider raised ADC in normal-appearing WM as being a “pre-leukoaraiosis” state [28]. Therefore some of the microstructural lesions that explain elevated ADC may be reversible after revascularization.

The significance of ADC elevation in chronic cerebrovascular disease is unclear. It may reflect subtle chronic ischemic damages with vasogenic oedema, gliosis, demyelination and axonal loss [31–33]. Elevated ADC values have been correlated with increasing loss of WM axonal anisotropy, which is likely due to hypoperfusion as seen in pathological studies [31, 34, 35]. DTI in animal models has shown that increased radial diffusivity and axial diffusivity could correspond to myelin breakdown and axonal damages [36].Development of collaterals may impact on cellular-level mechanisms to reverse cell dysfunction and so, some of these microstructural lesions. The decrease of ADC may be related to decreased oedema related to the improved functioning of the haematocencephalic barrier and myelin, or axonal repair thanks due to better perfusion. However, we did not observe any significant modification of CVR on PWI. MRI may have been performed too early to detect significant improvement in vasoreactivity. Some studies have found that the maximum improvement in cerebral haemodynamics by indirect bypass surgery began soon after surgery and gradually reached a maximum at 3 months after surgery, with MTT (mean transit time) measurement [37]. However, Cheung et al. showed that improvement of CVR measured with PET continued after 6 months and was the highest point between 12 and 24 months following by-pass surgery [38]. This suggests the development of collaterals and modification in perfusion long after surgery. Following revascularization, ADC may change earlier than vascular reserve. Microstructural WM repair may need just minor improvement in baseline perfusion in contrast to CVR. CVR measurement in PWI may also not be adapted to problems regarding acquisition and calculation due to the anastomoses from external carotid. Other explanations may be the small sample size or the failure of burr-hole surgery to improve CVR of the deep white matter. A previous study did not detect significant improvement in CVR after indirect revascularization [38]. Vessel autoregulation is more effective in the cerebral arteries, and greater blood flow may be achieved after acetazolamide challenge by direct haemodynamic increase to the middle cerebral artery, rather than the collateral achieved by burr-holes [39]. Comparison of ADC with other techniques or parameters of perfusion could be of interest in MMA.

The real clinical significance of such ADC modifications is not fully known. We need long-term follow-up studies to accurately assess the risk of recurrence of strokes in these patients. However, there was no recurrence of stroke during a 6-month follow-up, which suggests that this surgery is efficient. Moreover, 60% of our patients had improvements in cognitive results, as assessed in at least two tests. Overall TMT B-scores had increased, indicating improved cognitive flexibility. In adults with MMA, case reports have suggested potential improvement in cognitive functions post-surgery [19–21]. Yanagihara et al. were in agreement, reporting improvements in executive function in 31% of patients and no change in 25% at 2 months after by-pass, correlated with the chronic increase of cerebral blood flow [22]. Conversely, a bigger study of 84 patients showed improvements in only 11% of patients after a direct bypass; however, the surgery did not use the same revascularization techniques and the definition of improvement was more restricted [40]. We did not find any direct correlation between ADC change and improved cognition. Our small sample size involved a lack of statistical strength. In this context, the interpretation of these results is questionable. On the other hand, surgery may have affected cognition via other microstructural modifications like improvements in connectivity and the reorganization of white matter [41]. Nevertheless, it is not excluded that ADC may be considered as an intermediate criterion for exploring cognitive change. Previous studies have demonstrated an association between executive disorders and increased ADC, with a cut-off at around 800^10–6^ mm^2^/s [11, 17]. In our study, the mean ADC values in frontal normal-appearing WM was 789 ^10–6^ mm^2^/s after surgery.

Cognitive flexibility has been shown to be one of the most impaired processes [15]. Our ROIs were located in the centrum semiovale, on the pathways of tracts from prefrontal and posterior cingulate cortices towards deep grey nuclei. Increased ADC in these WM areas may be related to microstructural lesions that disrupt these tracts. TMT B, measuring flexibility, explores a part of these connecting tracts, so the potential reversibility of ADC values may explain some cognitive improvement after surgery [18]. This could explain the detectable impact of revascularization. No changes in other cognitive processes were observed when the entire population was considered. This may be due to a lack of statistical strength or the fact that the neuropsychological assessment was conducted too soon. However, it is important to note that, like Zeifert et al.’s study in which 75% of patients had unchanged cognition, our other scores did not worsen which is in contrast to what is expected in the natural history of MMA [40]. Revascularization surgery does not lead to a decline in cognitive functions. Indirect revascularization may stabilise conditions in some cases and improve some executive functions [22, 41].

These preliminary results provide evidence that microstructural lesions in frontal WM tracts that may be related to chronic frontal hypoperfusion seem to be involved in cognitive impairment in MMA. In line with this, Kazumata et al. showed with diffusion tensor imaging that fractional anisotropy and mean diffusivity of these tracts correlated with executive functions, suggesting the importance of microstructural damages of WM [18]. Another previous study demonstrated an association between TMT B scores and white matter disease in adults with MMA [42]. These results sustain the hypothesis of a “disconnection of WM projections from cortical areas”. Performing surgery before severe white matter disease develops may be useful in reversing some cognitive disorders.

Our study has several limitations. Firstly, these results should be interpreted with caution because of the small sample size, notably concerning neuropsychological evaluations. We cannot exclude that some improvement was related to a test--retest effect. Moreover, the improvements in some tests were not necessarily clinically significant because the results remained largely under normal Z-score. We used one surgical strategy in this study that focused on the frontal areas because we selected patients with predominant frontal hypoperfusion. Other techniques, particularly when combined, may yield differing results. Angiogenesis may be better achieved with combined bypass surgery. Larger studies comparing different strategies are necessary. Concerning ADC, healthy controls with a “normal” ADC value and patients with MMA that had not undergone surgery were not included. However, each patient was his own control with frontal versus posterior ADC measurements. Moreover, our ADC values remained abnormally high after surgery compared to the ADC values of 630--690^10 -6^ mm^2^/s found in healthy subjects [28, 43]. Finally, the relationship between ADC change and improvement of perfusion was indirect. We failed to demonstrate a significant improvement on cerebral blood volume and CVR in the whole population.

## Conclusions

Burr-hole surgery in adult patients with MMA and frontal hypoperfusion is followed by a decrease in ADC values that tended to normalize; in frontal normal-appearing WM. Change in ADC may indirectly reflect the improvement in cerebral perfusion post-surgery. Furthermore, burr hole surgery seems to improve cognitive flexibility. Some of the cognitive disorders associated with MMA may be explained by WM lesions and may be reversible following revascularization. This means that ADC measurement could be a promising tool for potentially exploring reversible microstructural damage in WM related to hypoperfusion and cognitive change.

List of abbreviations: ADC (apparent diffusion coefficient); CVR (cerebrovascular reserve); DSA (digital substracted angiography); DTI (diffusion tensor images); DWI (diffusion weighted images); FLAIR (Fluid-Attenuated Inversion Recovery); FOV (Field of View); MMA (moyamoya angiopathy); MRI (magnetic resonance imaging); MTT (mean transit time); NEX (Excitations Number); PET (positon emission tomography); PWI (perfusion weighted images); ROI (regions of interest); TE (Echo Time); TMT B (Trail Making Test part B); WM (white matter); TOF (circle of Willis Time Of Flight Magnetic Resonance Angiography); TR (Repetition Time); T1 G (Gadolinium-enhanced T1-weighted imaging); T2 (T2-weighted imaging); T2 GRE (T2 Gradient Echo);

## Data Availability

The dataset used and analysed during the current study are not publicly available due to French personal protection laws, but are available from the corresponding author upon reasonable request.
